# Special Issue “Multidrug-Resistant Bacteria in the Environment, Their Resistance and Transfer Mechanisms”

**DOI:** 10.3390/microorganisms11040981

**Published:** 2023-04-10

**Authors:** Elisabeth Grohmann

**Affiliations:** Faculty of Life Sciences and Technology, Department of Microbiology, Berliner Hochschule für Technik, 13347 Berlin, Germany; egrohmann@bht-berlin.de; Tel.: +49-(0)30-4504-3942

Multidrug-resistant bacteria are an emerging issue which is not restricted to clinics and the health care sector, but is increasingly affecting the environment. Multidrug-resistant bacteria are released into the environment via wastewater, improperly inactivated or disposed of waste and by applying organic fertilizers to agricultural fields, etc. The released bacteria are able to survive or even thrive in the environment and transfer their antibiotic resistance genes (ARG) to the autochthonous microbiome in aquatic environments, soils or even to plants/crops consumed by humans. In some instances, they die, releasing their DNA, which is then taken up by other bacteria in the environment. The latter mechanism is denominated transformation. The bacteria which can take up foreign “naked” DNA from the environment are naturally competent. In the first case, in which the released bacteria can survive in the environment, the major mechanism driving the dissemination of ARGs is conjugative transfer. The key players of this mechanism are conjugative plasmids, mobilizable plasmids, and very likely also integrative conjugative elements (ICEs) and genomic or pathogenicity islands [1,2,3]. The dissemination of resistance factors is facilitated, on one hand, by a high diversity of bacteria, which is a general feature of soils and aquatic environments, and on the other hand, by the availability of solid surfaces which can be easily colonized by bacteria [4,5]. Bacteria form surface-attached microbial communities, so-called biofilms in which different bacteria come into close contact with each other. Biofilms facilitate the gene exchange among diverse bacteria. They are considered hotspots of horizontal gene transfer [1,2,3,6].

In this Special Issue, eight articles, five research articles, one research communication, and two review articles addressed the main issues relating to the topic of the Special Issue. The paper of Korotetskiy and coworkers analyzed whole-genome sequences of pathogenic Gram-positive and Gram-negative isolates from the same hospital environment to elucidate evolutionary trends associated with horizontal gene transfer, mutations and DNA methylation patterns. They concluded that genotyping through third-generation sequencing and determination of patterns of genome methylation can be instrumental for monitoring the distribution of clonal lines of pathogens due to methylation patterns associated with specific pathogens [7]. Van Wonterghem et al. investigated the effect of *Escherichia coli* host factors on the transfer frequency of the broad-host-range plasmid pKJK10. They confirmed the role of the genes *fliF*, *fliK*, *kefB* and *ucpA* in the donor ability of conjugative elements by validating defects in the conjugation efficiency of the corresponding *E. coli* strain single-gene deletion mutants. Based on the cellular functions of these genes, the authors suggested that motility and energy supply, as well as the intracellular pH or salinity of the donor strain strongly affect the efficiency of plasmid transfer. Thus, the work of Van Wonterghem and coworkers could facilitate the search for targets for the development of conjugation inhibitors, which could be administered together with antibiotics to treat bacterial infections more effectively [8].

Hernández Gómez et al. studied the microbial community of commercial Mexican chili powder and its resistome. *Bacillaceae* were the most abundant family present in the commercial chili powders. Antibiotic resistance profiles of nosocomial pathogens detected in the chili samples revealed the presence of extended spectrum beta lactamases (ESBLs) and Metallo-beta-lactamases. These findings may facilitate developing procedures for microbial monitoring during chili powder production [9].

Colgan et al. and Werner et al. investigated the effect of different types of manure and compost generated by thermophilic composting of human excreta amended by green cuttings and sawdust on the composition of the bacterial community, abundance of bacterial pathogens and ARGs, respectively. The first study showed that the type of ARGs which persisted in soils was strongly dependent on the origin of the manure (cattle manure versus swine manure). They concluded that the distinct temporal dynamics of the resistance gene distributions in soil for each manure type could be used to develop potential mitigation strategies [10]. Werner and coworkers found low concentrations of beta-lactamase genes in samples prior to, during and after thermophilic composting, as well as a decline in the horizontal gene transfer marker genes, *intI1* and *korB,* from the start to the end of composting. Thus, thermophilic composting can decrease the horizontal spread of ARGs and could be a suitable treatment for the recycling of human excreta [11].

Miguel-Arribas and coworkers studied the presence of antitermination systems in conjugation operons of different conjugative plasmids of Gram-positive origin. These antitermination systems allow differential regulation of subsets of conjugation genes. Thus, they are involved in fine tuning of the expression of distinct components of the conjugation systems. Interestingly, the Inc18 family of Gram-positive broad-host-range conjugative plasmids does not contain an antitermination system. The possible advantages of the lack of an antitermination system in context of a broader conjugative host range of plasmids lacking such a system have been discussed [12].

Rozman and coworkers performed a systematic literature review on the reduced susceptibility and increased resistance of bacteria against disinfectants. They showed that reduced susceptibility to disinfectants and problems potentially related to antibiotic resistance in clinically relevant bacterial strains are increasing. The authors recommended the rotation of disinfectants, where one disinfectant should be replaced by another that has a different mechanism of action. In addition, they pointed out the necessity of developing and adopting strategies to control disinfectant resistance development [13]. In their review, Treskova et al. summarized the current research on antibiotic resistance in the environment conducted in Austria, Germany and Switzerland. They concluded that unified protocols for isolate collection, selecting sampling sites, and susceptibility testing are urgently required to provide results that can be compared between the studies. Epidemiological, environmental, and ecological factors must be considered in surveys of the environmental dissemination of antibiotic resistance. In addition, Treskova et al. pointed out the need for a ONE Health approach in all epidemiological studies analyzing antibiotic resistance [14].

In summary, there is an urgent need for an in-depth understanding of the diverse horizontal transfer mechanisms and their contribution to the spread of ARGs to develop efficient mitigation strategies. Moreover, innovative strategies must soon be developed to minimize the release of antibiotic resistant bacteria into the environment to avoid risking the efficient antibiotic treatment of bacterial infections.
